# Efficient Heuristics for Structure Learning of *k*-Dependence Bayesian Classifier

**DOI:** 10.3390/e20120897

**Published:** 2018-11-22

**Authors:** Yang Liu, Limin Wang, Minghui Sun

**Affiliations:** 1 Key Laboratory of Symbolic Computation and Knowledge Engineering of Ministry of Education, Jilin University, Changchun 130012, China; 2 College of Computer Science and Technology, Jilin University, Changchun 130012, China

**Keywords:** *k*-dependence Bayesian classifier, minimal-redundancy-maximal-relevance analysis, discriminative model selection

## Abstract

The rapid growth in data makes the quest for highly scalable learners a popular one. To achieve the trade-off between structure complexity and classification accuracy, the *k*-dependence Bayesian classifier (KDB) allows to represent different number of interdependencies for different data sizes. In this paper, we proposed two methods to improve the classification performance of KDB. Firstly, we use the minimal-redundancy-maximal-relevance analysis, which sorts the predictive features to identify redundant ones. Then, we propose an improved discriminative model selection to select an optimal sub-model by removing redundant features and arcs in the Bayesian network. Experimental results on 40 UCI datasets demonstrate that these two techniques are complementary and the proposed algorithm achieves competitive classification performance, and less classification time than other state-of-the-art Bayesian network classifiers like tree-augmented naive Bayes and averaged one-dependence estimators.

## 1. Introduction

In machine learning, classification is one of the most important tasks that predicts the unknown class labels according to some known evidence or labeled training samples. Bayesian network classifiers (BNCs) [1,2] provide a classical method to implement classification decision based on the probability framework. In general, BNCs consist of two parts, i.e., B=<G,Θ>. The network structure G is a directed acyclic graph (DAG). Nodes in G represent stochastic variables or features, arc $X_{i}\to X_{j}$ denotes probabilistic dependency relationships between these two features and $X_{i}$ is one of immediate parent nodes of $X_{j}$, i.e., $X_{i}\in Pa\left(X_{j}\right)$. Parameter Θ quantitatively describes this dependency. Let each instance **x** be characterized with *n* values $\left\{x_{1},\cdots ,x_{n}\right\}$ for features $\left\{X_{1},\cdots ,X_{n}\right\}$, and class label $c\in \{c_{1},\cdots ,c_{m}\}$ is the value of class variable *C*. Θ contains conditional probability tables $\theta_{x_{i}|Pa\left(x_{i}\right)}=p_{B}\left(x_{i}|Pa\left(x_{i}\right)\right)$ for each feature.

According to Bayesian theorem [3], BNC makes the classification decision in the following way:

(1) $$\arg\max_{C}p\left(c|x\right)=\arg\max_{C}\frac{p(x,c)}{p(x)}\propto \arg\max_{C}p\left(x,c\right)$$

According to the chain rule of joint probability distribution [1], p(x,c) can be calculated as follows:

(2) $$p\left(x,c\right)=p\left(c\right)p\left(x_{1}|c\right)p\left(x_{2}|x_{1},c\right)\cdots p\left(x_{n}|x_{1},x_{2},\ldots ,x_{n-1},c\right)=p\left(c\right)\prod_{i=1}^{n}p\left(x_{i}|Pa\left(x_{i}\right),c\right).$$

In this paper, we mainly focus on the restricted BNCs, which suppose that each feature is directly dependent on the class variable *C* and *C* does not have any parents. In this paper, we mainly focus on the restricted BNCs, which require that the class variable *C* be a parent of every feature and no feature be the parent of *C*.

The *k*-dependence Bayesian classifier (KDB) is one of the famous restricted BNCs [4]. To achieve the trade-off between structure complexity and classification accuracy, KDB allows to represent different number of interdependencies for different data sizes. During the learning procedure of KDB, it utilizes mutual information between features and class variable to rank and sort all features first. This sorting method gives priority to the features with high relevance between features and class. Feature $X_{i}$ may be a possible parent feature of $X_{j}$ if $X_{i}$ ranks before $X_{j}$, not the other way around. Then conditional mutual information between features is used to measure and select significant conditional dependencies. The dependency relationships between features and class, and that between different features, are considered in different learning phases. Obviously, some independent features with high mutual information value may achieve higher rank but demonstrate weak conditional dependencies. To address this issue, Peng et al. [5] propose a first-order incremental feature selection method based on minimal-redundancy-maximal-relevance (mRMR) criterion, which takes into account the maximal relevance between features and class, meanwhile considering the minimal redundancy between features. Its effectiveness has not been proved in the context of KDB.

The structure complexity will increase exponentially as the number of features increases. The features that rank at the end of the order are the least relevant to classification and may be disregarded. Regular KDB does not consider the negative effect caused by redundant features, which may bias the classification results. Many researchers have recognized that using a heuristic wrapper approach to delete redundant features helps minimize zero-one loss on the training samples [6,7,8]. Martínez et al. [9] propose discriminative model selection to select an optimal KDB sub-model which contains feature subset with necessary features. The resulting algorithm not only has the competitive classification performance of generative learning, but also has the excellent expressive power of discriminative learning. At each iteration for model selection, any feature $X_{i}$ in the order should have *k* parent features if i>k as KDB defines. However, the dependencies between $X_{i}$ at the end of the order and the other feature $X_{j}$ (1≤j≤n,i≠j) may be very weak, and these two features can be assumed to be independent. That is, the dependencies between $X_{i}$ and $X_{j}$ may be redundant.

In this paper, we will investigate the feasibility of applying discriminative model selection to remove redundant features and dependencies, and the interoperability of mRMR analysis and discriminative model selection. Section 2 reviews the state-of-the-art restricted BNCs, including naive Bayes (NB), tree-augmented naive Bayes (TAN) and especially KDB. In Section 3 we present the theoretical justification of our proposed algorithm, mRMR-based KDB with discriminative model selection (MMKDB).Section 4 presents a detailed analysis of the experimental results. Finally, we present conclusions in Section 5.

## 2. Restricted Bayesian Network Classifiers

The classification task in a BNC can be separated into two subtasks, structure learning and parameter learning. The former is to identify the structure of the network, and the latter is to calculate the probability distribution for a given network structure. In the following discussion, we will review some state-of-the-art BNCs from the perspective of structure learning and parameter learning.

NB is the simplest BNC [10,11], since the features are assumed to be conditionally independent given the class variable. The formula of joint probability p(x,c) is presented as follows:

(3) $$p\left(x,c\right)=p\left(c\right)\prod_{i=1}^{n}p\left(x_{i}|c\right).$$

**Figure 1:**
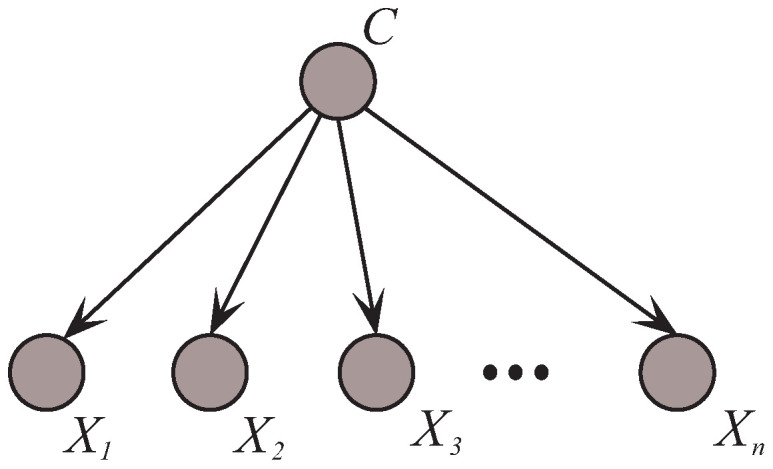
The topology structure of NB.

Note that, for NB, the parameter learning only involves the learning of the probability p(c) and the conditional probability $p(x_{i}|c)$, and the structure learning is not necessary since NB has a definite structure as shown in Figure 1. However, features may be interrelated in practice. Therefore, many researchers have exploited methods to alleviate the conditional independence assumption of NB [12,13,14]. It is worthwhile to mention that, Webb et al. [15] present a new approach, named averaged one-dependence estimators (AODE), to weaken the feature independence assumption by averaging all of the constrained class of classifiers. The class of all such classifiers has all other features depend on a common feature and the class variable.

TAN [1] is an extension of NB. It uses a variant of the Chow-Liu algorithm [16] to construct the Bayesian network, and it utilizes conditional mutual information

(4) $$I\left(X_{i};X_{j}|C\right)=\sum_{x_{i}\in X_{i}}\sum_{x_{j}\in X_{j}}\sum_{c\in C}p\left(x_{i},x_{j},c\right)log_{2}\frac{p(x_{i},x_{j}|c)}{p\left(x_{i}|c\right)p\left(x_{j}|c\right)}$$

to find a maximum weighted spanning tree. Additional arcs between features are allowed, i.e., dependencies between features can be captured. Each feature in the network has at most one other feature as its parents, except a single feature (the root of the tree), which has only the class variable as its parent. TAN alleviates some of conditional independence assumption of NB and, thus, improves its prediction accuracy at the cost of adding its structure complexity. The joint probability of TAN is calculated by:

(5) $$p\left(x,c\right)=p\left(c\right)\prod_{i=1}^{n}p\left(x_{i}|x_{j},c\right).$$

where $X_{j}$ is the parent of $X_{i}$ in the tree structure.

**Figure 2:**
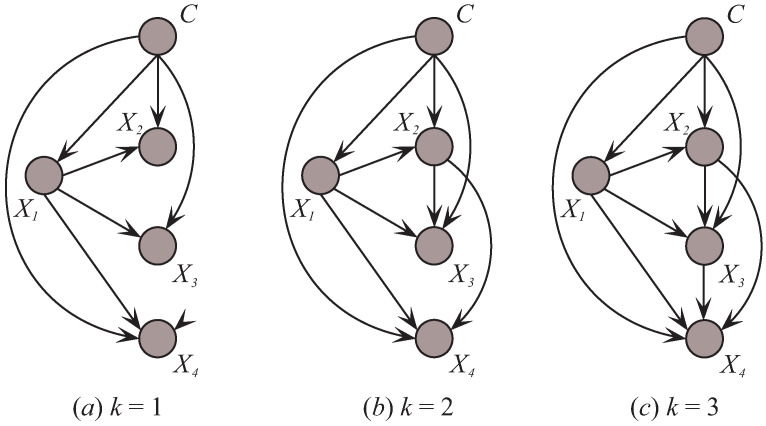
Some examples of corresponding structures of KDB classifiers when given different *k* values.

KDB is another classical improvement to NB [4]. It allows for most *k* features to be the parents for each feature. In this sense, NB is a 0-dependence BNC and TAN is a one-dependence BNC. In the real-world domains we find that modeling feature dependencies very often improves classification performance. This is especially true for the KDB, with respect to lower value of *k*, larger value of *k* may helps to improve the classification accuracy [4]. Two passes are required for KDB to learn over the training examples. Structure learning is the first pass. Algorithm 1 depicts the structure learning process of KDB. Parameter learning is the second pass. According to the Bayesian network obtained from the former pass, the joint probability of KDB for each instance can be calculated by:

(6) $$p\left(x,c\right)=p\left(c\right)\prod_{i=1}^{n}p\left(x_{i}|Pa\left(x_{i}\right),c\right).$$

where $Pa(x_{i})$ denotes the parents of $X_{i}$ in the structure. Suppose there is an ordered feature set $\left\{X_{1},X_{2},X_{3},X_{4}\right\}$, we give some examples of corresponding structures of KDB classifiers in Figure 2 when given different *k* values. Corresponding joint probability distributions are shown in Table 1.

**Algorithm 1:** Structure learning process of KDB.

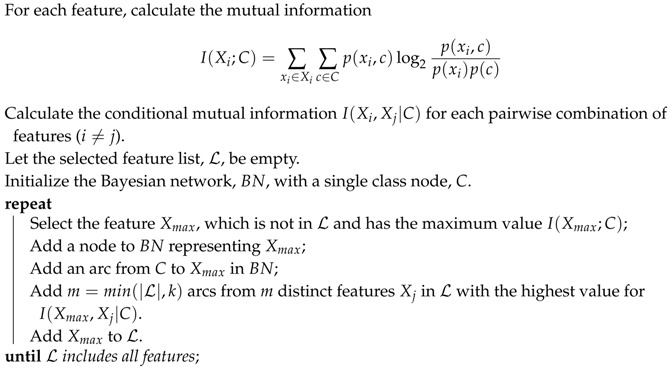



## 3. The mRMR-Based KDB with the Discriminative Model Selection

To elaborate our motivations for doing selection based on mRMR criterion and discriminative model selection in the context of the KDB classifier, we consider two extreme examples of constructing BNCs over two sets of features. The first feature set contains two perfectly correlated features $X_{i}$ and $X_{j}$, where $X_{i}$ is an exact copy of $X_{j}$. Both $X_{i}$ and $X_{j}$ will be included in the network structure of KDB, that is, $X_{i}$ (or $X_{j}$ alternatively) will have twice the influence of the other features, which may strongly bias the performance of the classifier. A possible way to improve the classification performance is to eliminate one of the features $\left\{X_{i},X_{j}\right\}$ from the feature set and to construct the classifier over the reduced set of features. The ordered feature set $\left\{X_{a},X_{b},X_{c}\right\}$ is the second extreme example and contains non-redundant features to construct a KDB with k=2. Suppose that the values of $I(X_{c};X_{a}|C)$ and $I(X_{c};X_{b}|C)$ are respectively 0.99 and 0.0001. As KDB defines, feature $X_{c}$ should select $X_{a}$ and $X_{b}$ as its parent features in any case. This naturally results in a redundant dependency between $X_{c}$ and $X_{b}$, which may lead to negative effects on the classification performance of KDB and increases the risk of over-fitting at a certain extent.

Therefore, we utilize the sorting method based on an mRMR criterion to identify possible redundant features and discriminative model selection to achieve the aim of removing the redundant features or conditional interdependencies. The usual feature selection based on mutual information in KDB intends to select features that are independent of each other. Instead, the mRMR method tries to select a feature that minimizes the redundancy and maximizes the relevance. As argued by Peng et al. [5], for real data, the features selected in this way will have more or less correlation with each other and the joint effect of these features can lead to very good classification accuracy.

Let *S* denote the feature set and |S| is the cardinality of *S*. Given a feature set $X=\{X_{1},X_{2},\ldots ,X_{n}\}$ and a class variable *C*, in order to make sure that the selected feature subset is the most appropriate one, two conditions should be met. The first one is the minimum redundancy condition [5]:

(7) $$Min\;R\left(S\right)\;,where\;R\left(S\right)=\frac{1}{{\left|S\right|}^{2}}\sum_{X_{i},X_{j}\in S}I\left(X_{i};X_{j}\right)$$

where *R* represents the level of redundancy between features.

And the other one is the maximum relevancy condition [5]:

(8) $$Max\;D\left(S\right)\;,where\;D\left(S\right)=\frac{1}{\left|S\right|}\sum_{X_{i}\in S}I\left(X_{i};C\right)$$

where *D* represents the level of relevancy between feature and class variable.

There are two combinations of these two conditions, named MID (Mutual Information Difference) and MIQ (Mutual Information Quotient) [17], which balance the two objectives, maximum relevance and minimum redundancy, in different ways as follows:

(9) MID(S)=max(D(S)−R(S))

(10) MIQ(S)=max(D(S)/R(S))

As argued by Gulgezen et al. [18], MID produces more stable feature subsets, so in this paper we choose MID as the criterion. Suppose there exists a selected feature subset $S_{m-1}$, which consists of m−1 features, then the *m*-th feature can be determined by following equation:

(11) $$\nabla_{MID}\left(X_{j}\right)=max\left\{I\left(X_{j};C\right)-\frac{1}{m-1}\sum_{X_{i}\in S_{m-1}}I\left(X_{i};X_{j}\right)\right\}$$

where $X_{j}\in S-S_{m-1}$.

The feature selection based on mRMR criterion utilizes forward selection strategy, it starts with an empty feature set L and then iteratively add one feature into the L at a time by Equation (11). Sorting all features in this way, we consider the feature subsets $\left\{X_{1},X_{2},\ldots ,X_{i}\right\}$, 1≤i≤n, each feature subset contains *i* ordered features. That is, for *n* features there are *n* alternative feature subsets that could be explored for our proposed algorithm.

From Equation (6) we can observe that the joint probability p(x,c) can be considered as the product of a set of conditional probabilities $p(x_{i}|Pa\left(x_{i}\right),c)$. This means that we can build a model space by using a nested method, each model can be built upon the previous one. For an instance $x=(x_{1},x_{2},\ldots ,x_{n})$, as Table 2 shows, the joint probability $p{\left(x,c\right)}_{2}$ is obtained by multiplying the conditional probability of feature $X_{2}$ (i.e., $p(x_{2}|Pa\left(x_{2}\right),c)$) to $p{\left(x,c\right)}_{1}$ and the joint probability $p{\left(x,c\right)}_{3}$ is obtained by multiplying the conditional probability of feature $X_{3}$ (i.e., $p(x_{3}|Pa\left(x_{3}\right),c)$) to $p{\left(x,c\right)}_{2}$. That is to say, if the model $p{\left(x,c\right)}_{i}$ has been built, it is not necessary to repeat the process of structure learning with feature set $\left\{X_{1},X_{2},\ldots ,X_{i}\right\}$ for the model $p{\left(x,c\right)}_{i+1}$. We only need to find parents of the feature $X_{i+1}$ in the BN and then multiply the conditional probability $p(x_{i+1}|Pa\left(x_{i+1}\right),c)$ with the joint probability $p{\left(x,c\right)}_{i}$ (which has been learnt in the previous model $p{\left(x,c\right)}_{i}$) to obtain the model $p{\left(x,c\right)}_{i+1}$. The discriminative model selection framework is derived from the chain rule of BNCs’ joint probability, it firstly constructs a space of sub-models, and then selects the best sub-model by the evaluation function to achieve our purpose in feature selection.

**Figure 3:**
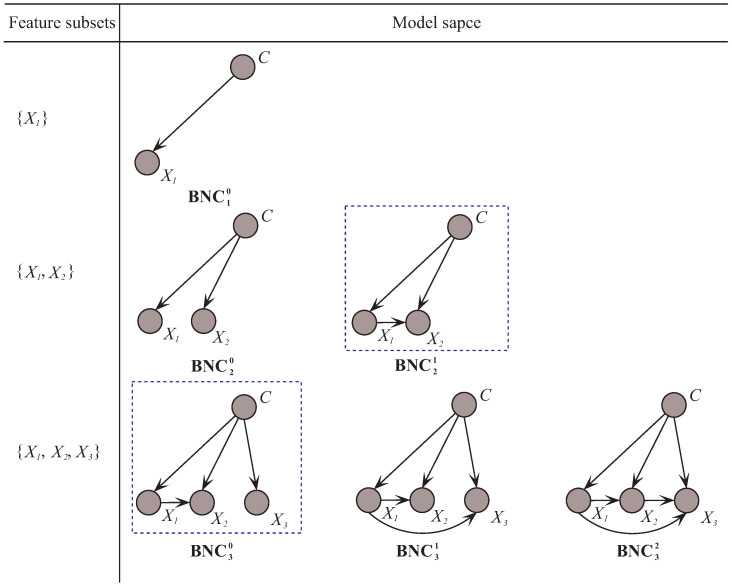
The examples of corresponding model space of MMKDB for three feature subsets when k=2.

Based on the above observations and discussions, we further improve the framework of discriminative model selection from the view of feature dependencies. To make the idea of the improved framework of discriminative model selection clear in KDB, we restrict that at most two features can be the parents for each feature in the following discussion. As Figure 3 shows, for feature subset $\left\{X_{1},X_{2},X_{3}\right\}$, the corresponding model space of our proposed algorithm MMKDB is composed of BNC${}_{3}^{0}$, BNC${}_{3}^{1}$ and BNC${}_{3}^{2}$. The only difference of these three BNCs is the number of parents for feature $X_{3}$. We employ the conditional mutual information to assign 0, 1 or 2 features to $X_{3}$ as parents, respectively. Note that all BNCs with $\left\{X_{1},X_{2},X_{3}\right\}$ are built upon BNC${}_{2}^{1}$, which is the best BNC for feature subset $\left\{X_{1},X_{2}\right\}$ and selected by using an evaluation function. Similarly, BNC${}_{3}^{0}$, BNC${}_{3}^{1}$ and BNC${}_{3}^{2}$ also need to be evaluated the classification performance to select the best one. In this way, we can remove not only redundant features but also redundant dependencies between them. That is to say, at each iteration for model selection, any feature $X_{i}$ should have $k^{\prime }$ parent features, where $0\leq k^{\prime }\leq k$ if i>k. Note that we employ the root mean squared error (RMSE) [19] as the evaluation function in the procedure of discriminative model selection, which is an effective measure of probability estimates:

(12) $$RMSE=\sqrt{\frac{1}{t}\sum_{x\in D}{\left(1-p\left(\hat{c}|x\right)\right)}^{2}}$$

where D is the training set, *t* is the number of training examples, $\hat{c}$ is the true class label for the instance x, and $p(\hat{c}|x)$ is the estimated posterior probability of the true class given x.

It is worthwhile to note that, in order to avoid over-fitting of sub-models on training examples, we employ the leave-one-out cross-validation (LOOCV) [20] to evaluate the classification performance of each model. Kohavi et al. [21] propose an incremental method to refine the cross-validation. The traditional LOOCV for BNCs recomputes the joint probability of a new model over the training examples for each instance. Differently, the incremental cross-validation firstly calculates the total joint frequency counts for all training examples, and then when testing an instance, temporarily removing its counts from the total counts to calculate the joint probability of corresponding model.

**Figure 4:**
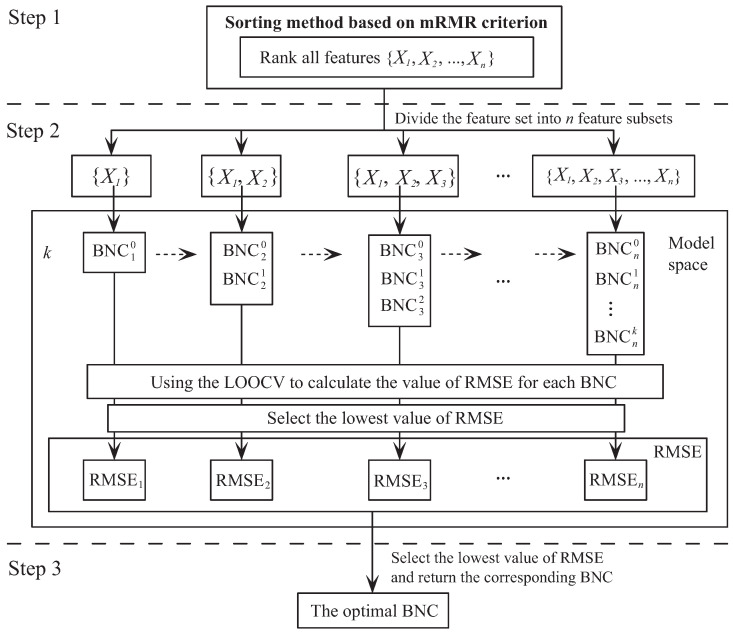
The schematic diagram of MMKDB.

Figure 4 presents the schematic diagram of our proposed algorithm MMKDB. Step 1 sets the order of features by the mRMR sorting method, computes the conditional mutual information between features and class variable through the training examples, and the ordered feature set would be divided into *n* feature subsets. Steps 2 and 3 correspond to the framework of discriminative model selection. All ordered feature subsets are introduced as the input to construct the corresponding BNCs. Sub-models containing features that rank ahead in the order would be built upon sub-models containing features ranks behind. These $\frac{n(k+2)}{2}$ sub-models form the model space. Each sub-model denotes as BNC${}_{s}^{r}$, where *s* is the number of feature subsets and *r* is the number of parents for feature $X_{s}$. In the model space, all sub-models are evaluated by using the LOOCV to calculate the values of RMSE through the training examples. According to the chain rule of BNCs’ joint probability, BNC${}_{s+1}$ needs to be built upon BNC${}_{s}$. Thus, only one sub-model that has the lowest value of RMSE would be selected for each feature subset. Finally, there are *n* alternative local optimum BNCs for *n* feature subsets. The optimal BNC would be selected from these *n* sub-models.

Based on the discussion presented above, we present the pseudo-codes of MMKDB in Algorithm 2. Calculating $I(X_{i};C)$ and $I(X_{i};X_{j})$ respectively need O(tcnv) and $O(tn^{2}v^{2})$ time, where *t* is the number of training examples, *c* is the number of classes, *n* is the number of features and *v* is the maximum number of possible values per feature. From Equation (11) we can infer that, if (n>c) the time complexity of step 2 in Algorithm 2 is $O(tn^{2}v^{2})$, or else O(tcnv). The procedure of computing the conditional mutual information needs $O(tcn^{2}v^{2})$ time. The space complexity of the table of joint frequencies of all combinations of *n* features values and the class label is $O(cn^{2}v^{2})$. Feature ordering needs O(nlogn) time and parent assignment for each feature needs $O(n^{2}\log n)$ time. Moreover, classify an instance using the selected sub-model only requires O(cnk) time. That is, the procedure of discriminative model selection needs $O(tcnk^{2})$ time. So the overall time complexity is $O(tcn^{2}v^{2}+tcnk^{2})$ for MMKDB and $O(tn^{2}v^{2})$ for KDB. This is an acceptable result, since *k* is a user-set parameter. That is, the time complexity of MMKDB scales linearly with the number of training examples, classes and features.

**Algorithm 2:** Algorithm MMKDB.

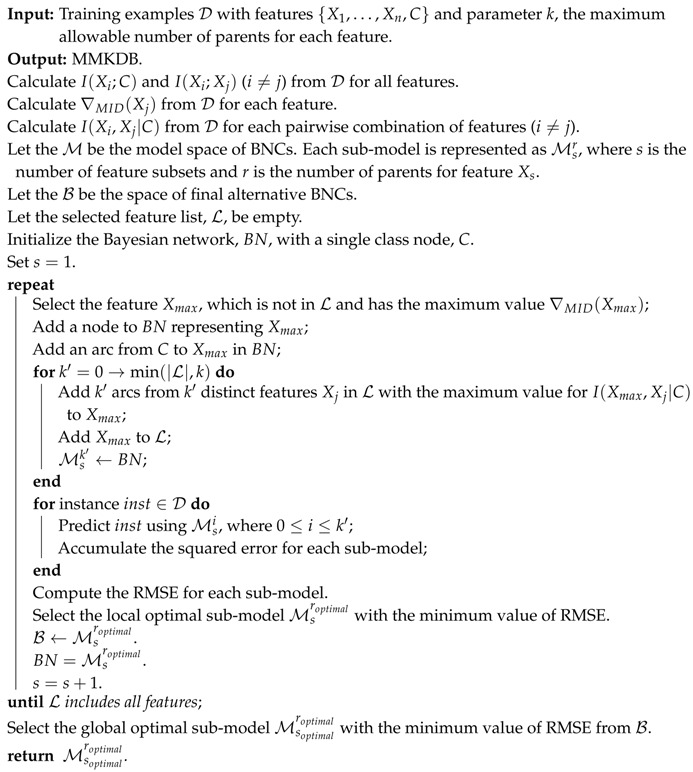



## 4. Experiments

We run the experiments on a C++ system (GCC 5.4.0) which is specially designed for BNCs. For KDB, with respect to lower value of *k*, larger value of *k* may helps to improve the classification accuracy. However, the restrictions of currently available hardware place some requirements on the software. The structure complexity and time complexity will increase exponentionally as *k* increases. When k=4, due to the amount of memory and CPU available the experimental results of MMKDB on some datasets cannot be achieved. Thus in the following experimental study the maximum value of *k* is 3.

In our experimental study, we gather a group of datasets from UCI machine learning repository [22]. These datasets are described in Table 3. Missing values are referred to as a distinct value. For each dataset, we discretize quantitative features using 5-bin equal frequency discretization, and we employ the *m*-estimation (m=1) [23,24] to smooth the probability estimates.

As a contrast, we also present respectively two extensional version of KDB as follows:

- KDB with the sorting method based on the mRMR criterion (MKDB).
- KDB with the discriminative model selection (MSKDB).

Note that, the experiments have been done by using 10 rounds of 10-fold cross-validation, and we employ the zero-one loss to evaluate classification accuracy of different algorithms [25]. Suppose that *c* is the predicted class label of an algorithm and $\hat{c}$ is the true class label, the value of zero-one loss is calculated as follow:

(13) $$\xi \left(c,\hat{c}\right)=1-\delta \left(c,\hat{c}\right)$$

where $\delta (c,\hat{c})=1$ if $c=\hat{c}$ and 0 otherwise.

The detailed zero-one loss results of all alternative algorithms are presented in Table A1 in the Appendix A. In order to give the experimental results an intuitive explanation, we employ the Win/Draw/Loss (W/D/L) records to summarize the number of datasets for different algorithms in the following three situations on a given evaluation function: a win represents an algorithm achieves significant advantages over the other one on a dataset, a loss indicates the opposite case and the draw suggests that these two algorithms perform comparably. Each entry compares the algorithm in the row against the one in the column. We regard a difference as significant between two algorithms if their outcomes of a one-tailed binomial sign test is less than 0.05.

### 4.1. Impact of Sorting Method Based on the mRMR Criterion and Discriminative Model Selection

In order to investigate the impact of sorting method based on the mRMR criterion, we present the W/D/L records when comparing the zero-one loss results of KDB and MKDB in Table 4. The only difference between KDB and MKDB is the sorting method of features, the former performs the sorting method based on the mutual information and the latter performs the one based on mRMR criterion. From Table 4 we can see that, MKDB achieves significant advantages over KDB and results in W/D/L of 12/25/3. This proves that the sorting method based on the mRMR criterion is superior to the one based on mutual information in KDB. Compared with KDB, there are only three datasets, i.e., Lung-Cancer, House-Votes-84 and Anneal, have higher results of zero-one loss over MKDB, which indicates that MKDB seldom performs worse than KDB, and for many datasets, it substantially improved the classification performance of KDB, such as, the datasets Adult, Dermatology, Labor and Hypo.

In order to explore the effect of discriminative model selection, we present the W/D/L records in terms of zero-one loss between KDB and MSKDB in Table 5. The only difference between these two algorithms is that MSKDB need an extra pass to perform the discriminative model selection through the training examples. As expected, MSKDB achieves lower zero-one loss results more often than KDB, for example, the decrease from 0.1926 to 0.0598 for the dataset Splice-C4.5. Note that MSKDB only performs worse than KDB on one dataset, i.e., Contact-Lenses. We argue that the lack of enough instances is the main reason why MSKDB performs not well on this dataset.

### 4.2. Comparison of MMKDB vs. KDB

According to the zero-one loss, the corresponding comparison with MMKDB and KDB is given in Table 6. Table 6 also presents the W/D/L records of MMKDB over MKDB and MSKDB. As we can see that MMKDB achieves significant advantages than KDB, MKDB and MSKDB, which indicates that the interoperability of mRMR analysis and discriminative model selection is feasible. To further demonstrate the performance of MMKDB over other algorithms, we employ the goal difference (GD) [26]. Suppose there are two classifiers *A* and *B*, the value of GD can be computed as follow:

(14) GD(A;B|T)=|win|−|loss|,

where T is the datasets, |win| and |loss| represent the number of datasets on which *A* performs better or worse than *B*, respectively.

**Figure 5:**
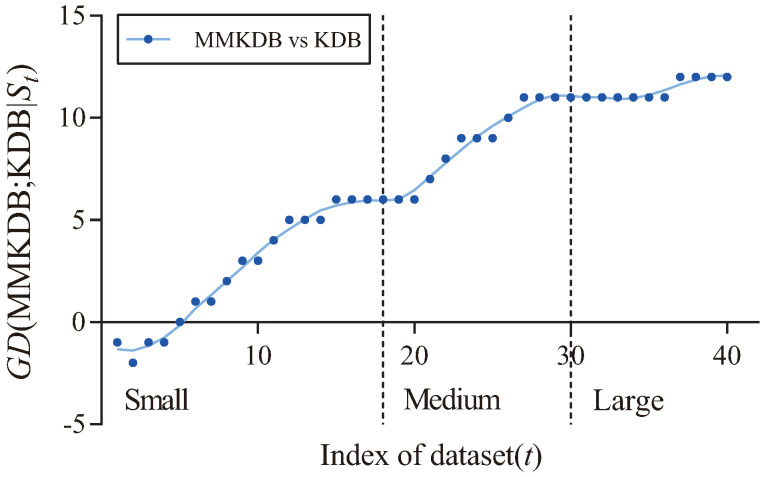
The fitting curve of GD(MMKDB; KDB$|S_{t})$ in terms of zero-one loss.

Figure 5 shows the fitting curve of GD(MMKDB; KDB$|S_{t})$ in terms of zero-one loss. The X-axis shows the indexes of different datasets, referred to as *t*, which correspond to that described in Table 3, and the Y-axis corresponds to the value of GD(MMKDB; KDB$|S_{t})$, where $S_{t}=\left\{D_{m}|m\leq t\right\}$ and $D_{m}$ is the dataset with index *m*. We categorize datasets according to their size. Datasets with instances ≤1000, >1000 and ≤10,000, >10,000 are represented as small, medium and large size, respectively. Two dotted lines divide the figure into three parts, each part is associated to the corresponding sizes of different datasets. From Figure 5 we can see a clear positive correlation between the values of GD(MMKDB; KDB$|S_{t})$ and the dataset size. As the size of datasets increases, MMKDB achieves significant advantages over KDB on small and medium datasets. When the number of instances >10,000, MMKDB has similar zero-one loss performance to KDB, but it speeds up classification time. Since MMKDB removes not only features but also dependencies between them may be redundant. Thus, we can come to the conclusion that, MMKDB not only retains the privileges of KDB, i.e., the capacity of high dependence representation and the model fitting ability on large datasets, but also improves the model fitting ability on small and medium datasets and enhances the classification efficiency on large datasets. That is, it proves the feasibility of applying discriminative model selection to remove redundant features and dependencies.

To further evaluate whether mRMR analysis and discriminative model selection are compatible and the extent to which applying both together improves the classification performance relative to applying each alone, we employ the relative zero-one loss ratio [27]. Given two classifiers *A* and *B*, the value of the relative zero-one loss ratio, referred to as $R_{Z}\left(\cdot \right)$, is calculated as follow:

(15) $$R_{Z}\left(A|B\right)=1-\frac{Z_{A}}{Z_{B}}$$

where Z denotes the zero-one loss, and $Z_{A(or\;B)}$ is the value of zero-one loss of classifier A(orB) on a dataset. The smaller ratio of $Z_{A}$ and $Z_{B}$, the higher value of $R_{Z}\left(A|B\right)$, and the better performance of *A*.

**Figure 6:**
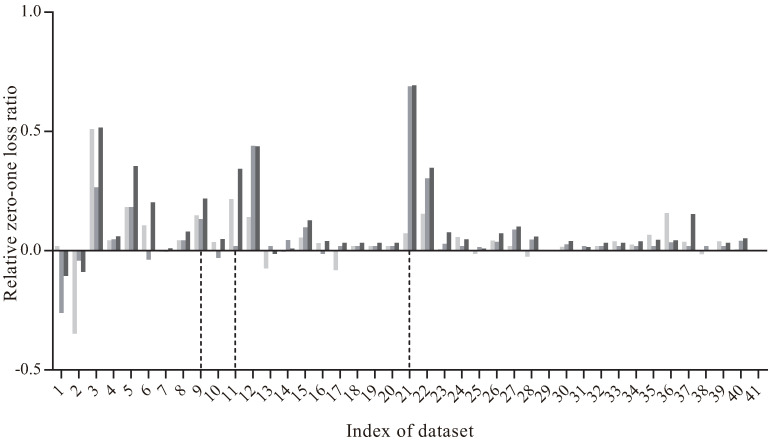
The comparison results of relative zero-one loss ratio between MKDB, MSKDB, MMKDB and KDB.

Figure 6 presents the comparison results of $R_{Z}\left(\cdot \right)$ between MKDB, MSKDB, MMKDB and KDB. The X-axis shows the index of dataset, and the Y-axis corresponds to the value of $R_{Z}\left(\cdot \right)$. As we can see, on the dataset Audio (No. 9), the values of $R_{Z}$(MKDB|KDB) and $R_{Z}$(MSKDB|KDB) are respectively 0.1486 and 0.1328. But when it comes to MMKDB, $R_{Z}$(MMKDB|KDB) is 0.2184, which is more higher than the former two results. There are also two extreme situations. For the dataset Dermatology (No. 11), $R_{Z}$(MKDB|KDB) is 0.2006 but $R_{Z}$(MSKDB|KDB) is 0.0198. That is, MKDB improves significantly with KDB but MSKDB does not. But nevertheless, the value of $R_{Z}$(MMKDB|KDB) on dataset Dermatology is 0.3482. Another extreme situations is just on the contrary, such as dataset Splice-C4.5 (No. 21), $R_{Z}$(MKDB|KDB) is 0.0729 and $R_{Z}$(MSKDB|KDB) is 0.6895, which are very unbalanced. However, $R_{Z}$(MMKDB|KDB) is 0.6936. That is, the value of $R_{Z}$(MMKDB|KDB) is always equally well to or better than $R_{Z}$(MKDB|KDB) and $R_{Z}$(MSKDB|KDB). Therefore, we can draw a conclusion that mRMR analysis and discriminative model selection are compatible in the framework of KDB.

**Figure 7:**
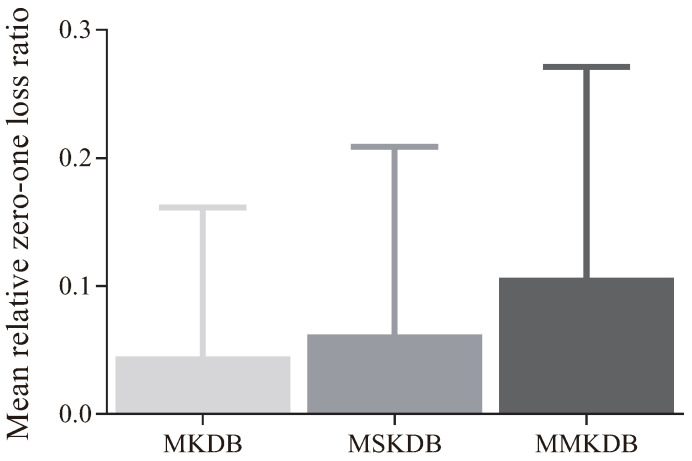
The comparison results of mean relative zero-one loss ratio between MKDB, MSKDB, MMKDB and KDB.

A more intuitive explanation is presented in Figure 7. Each bar represents the mean relative zero-one loss ratio of an algorithm to KDB on 40 datasets. As shown in Figure 7, the values of ${\bar{R}}_{Z}$(MKDB|KDB), ${\bar{R}}_{Z}$(MSKDB|KDB) and ${\bar{R}}_{Z}$(MMKDB|KDB) are respectively 0.0449, 0.0620 and 0.1006. That is, the average improved extent of MMKDB to KDB is obviously higher than MKDB and MSKDB. It proves that the interoperability of mRMR analysis and discriminative model selection is the major reason why applying both together improves the classification performance relative to applying each alone.

### 4.3. Comparison of MMKDB vs. NB, TAN and AODE

**Figure 8:**
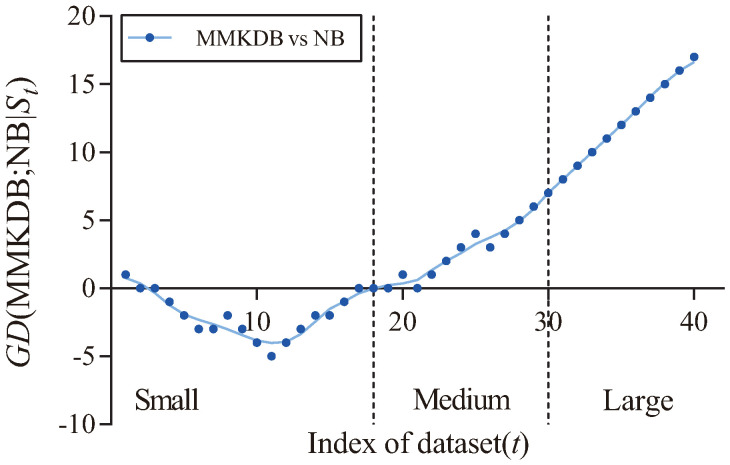
The fitting curve of GD(MMKDB; NB$|S_{t})$ in terms of zero-one loss.

**Figure 9:**
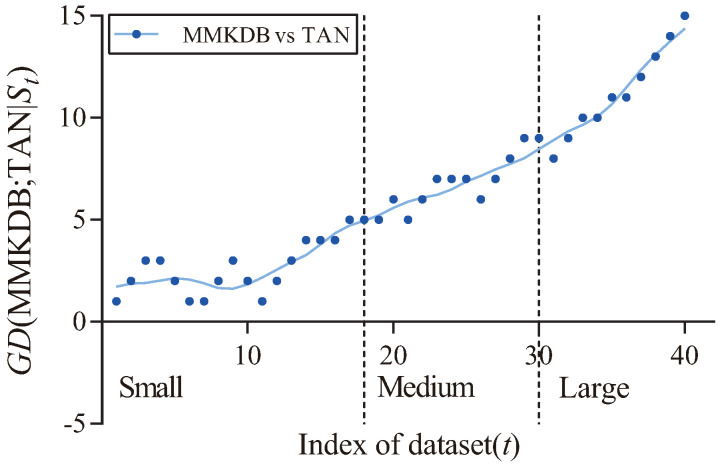
The fitting curve of GD(MMKDB; NB$|S_{t})$ in terms of zero-one loss.

Table 7 presents the corresponding W/D/L results. As we can see, the zero-one loss results of MMKDB are significantly better when compared to NB and TAN, MMKDB also achieves competitive classification performance over AODE. Figure 8 and Figure 9 respectively present the fitting curves of GD(MMKDB; NB$|S_{t})$ and GD(MMKDB; TAN$|S_{t})$ in terms of zero-one loss. As we can see that, MMKDB has similar performance to NB and TAN on small datasets. However, when the dataset size increased to 1000 instances (Led, No. 18), the prediction performance of MMKDB is obviously better than NB and TAN. That is, MMKDB achieves significant advantages over NB and TAN on medium and large datasets.

**Figure 10:**
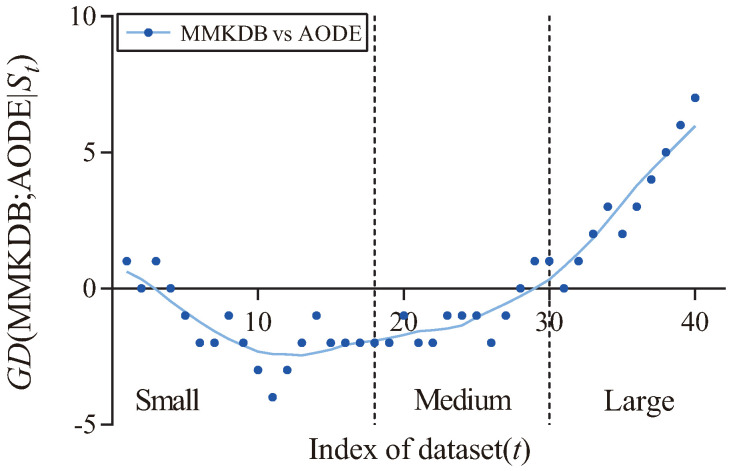
The fitting curve of GD(MMKDB; AODE$|S_{t})$ in terms of zero-one loss.

For one of the famous ensemble BNCs, AODE, Figure 10 presents the corresponding fitting curve of GD(MMKDB; AODE$|S_{t})$ in terms of zero-one loss. we can see that the values of GD decrease when the dataset size ≤1000, which means that MMKDB is very difficult to beat AODE on small datasets. When the dataset >1000 and ≤10,000, MMKDB has similar classification performance to AODE. This makes MMKDB a good substitute to AODE on medium datasets. Note that the fitting curve obviously turns upward when the dataset size >10,000 (the size of dataset Pendigits, No. 31). That is to say, the single BNC, MMKDB, achieves significant advantages over AODE on large datasets.

**Figure 11:**
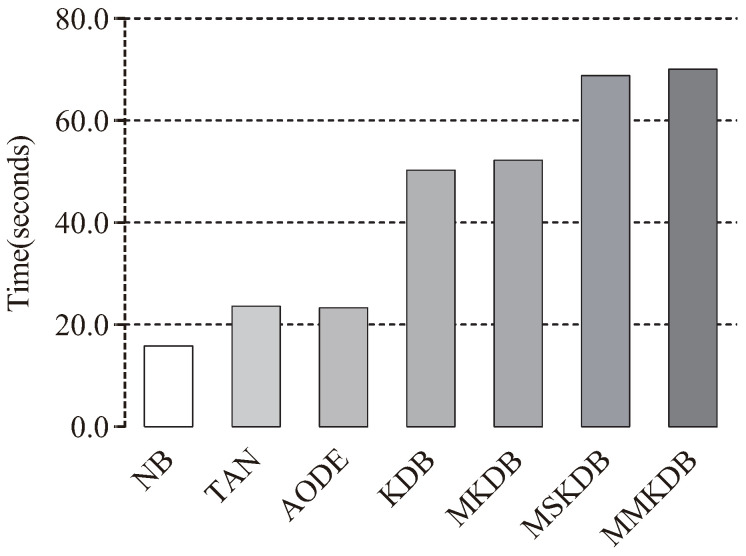
Training time comparisons of NB, TAN, AODE, KDB, MKDB, MSKDB and MMKDB.

**Figure 12:**
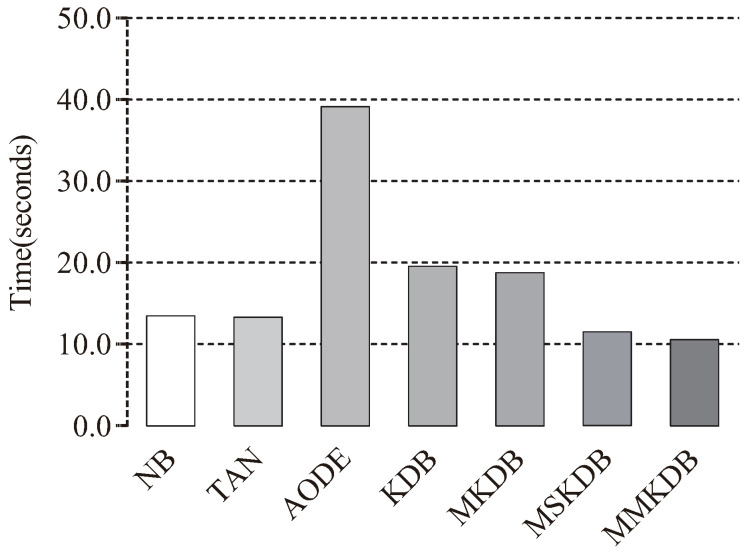
Classification time comparisons of NB, TAN, AODE, KDB, MKDB, MSKDB and MMKDB.

The training and classification time comparisons of NB, TAN, AODE, KDB, MKDB, MSKDB and MMKDB are shown in Figure 11 and Figure 12. Each bar depicts the sum of time on 40 datasets. Although, for most datasets our proposed algorithm MMKDB requires substantially more time for learning than other BNCs, such NB, TAN, AODE and KDB, while the classification time of MMKDB is the least. Note that the training and classification time of MSKDB is similar to MMKDB, and the training and classification time of MKDB is similar to KDB. AODE is an ensemble algorithm. Its classification time increases quadratically with the number of features, and hence much higher for other BNCs in Figure 12. In general, MMKDB saves about 42% of KDB’s classification time and greatly improves the classification performance of KDB at the cost of increasing less training time, and it also enjoys an even greater advantage at classification time compared to NB, TAN and AODE.

### 4.4. Global Comparison

In this section, we use the Friedman test for comparison of all alternative algorithms on 40 datasets to perform the significance test [28]. The Friedman test is a non-parametric measure, it can be computed as follows:

(16) $$F_{F}=\frac{\left(D-1\right)\chi_{F}^{2}}{D\left(g-1\right)-\chi_{F}^{2}}$$

and

(17) $$\chi_{F}^{2}=\frac{12D}{g(g+1)}\left(\sum_{i}R_{i}^{2}-\frac{g{\left(g+1\right)}^{2}}{4}\right)$$

where *g* is the number of alternative algorithms, *D* is the number of datasets and $R_{i}$ is the average rank of the *i*-th algorithm. The best performing algorithm getting the rank of 1, the second best rank 2, *…*. In case of ties, average ranks are assigned. The null hypothesis of the Friedman test is that there is no difference in average ranks. The detailed results of the average rank on 40 datasets are presented in Table A2 in the Appendix A. With 7 algorithms and 40 datasets, the Friedman test is distributed according to the *F* distribution with g−1=7−1=6 and (g−1)(D−1)=(7−1)×(40−1)=234 degrees of freedom. The critical value of F(6.234) for α=0.05 is 2.1375. The result of Friedman test for zero-one loss, $F_{F}=8.6308>2.1375$ with p<0.001. Hence, we reject the null-hypothesis. That is to say, the seven algorithms are not equivalent in terms of zero-one loss results.

**Figure 13:**
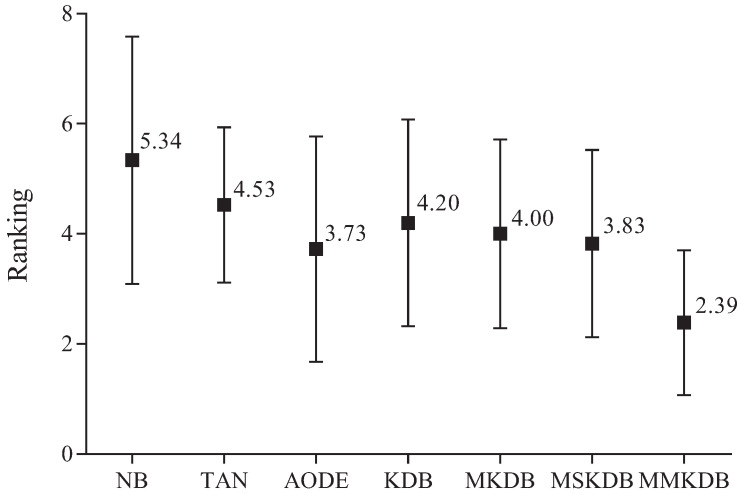
The results of ranking in terms of zero-one loss for all alternative algorithms.

Figure 13 presents the results of ranking in terms of zero-one loss for all alternative algorithms. The average ranks of different algorithms in terms of zero-one loss on all datasets are respectively {NB(5.34), TAN(4.53), AODE(3.73), KDB(4.20), MKDB(4.00), MSKDB(3.83), MMKDB(2.39)}. That is, the ranking of MMKDB is better than that of other algorithms, followed by AODE, MSKB, MKDB, KDB, TAN and NB.

**Figure 14:**
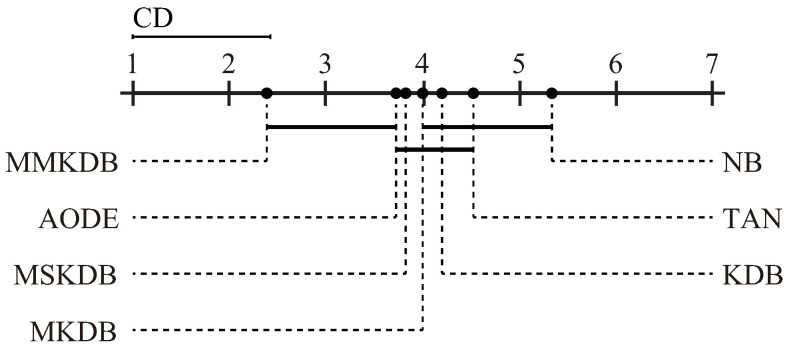
The results of Nemenyi test in terms of zero-one loss for all alternative algorithms.

In order to further explore which algorithm is significantly different to others, we also perform the Nemenyi test [29] shown in Figure 14. The algorithms are plotted on the dotted line on the basis of their average ranks, which are corresponding to the nodes on the top solid line. Critical Difference (CD) is also shown in the figure. The value of CD is calculated as follow:

(18) $$CD=q_{\alpha }\sqrt{\frac{g(g+1)}{6D}}$$

where the critical value $q_{\alpha }$ for α=0.05 and g=7 is 2.949. For α=0.05 with 7 algorithms and 40 datasets, CD = $2.949\times \sqrt{g\times (g+1)/(6\times D)}=2.949\times \sqrt{7\times (7+1)/(6\times 40)}=1.4245$. It is worthwhile to note that, the more leftward the position of algorithms on the black line, the lower the rank will be, and hence the better the performance. The algorithms are connected by a line if their differences are not significant. As the figure shows, NB, TAN, KDB and MKDB have equivalent mean rank. The mean rank of MMKDB is significantly lower than those of NB, TAN, KDB, MKDB and MSKDB. MMKDB also achieves lower mean ranks than AODE, but not significantly so.

## 5. Conclusions

KDB is a famous BNC with the capacity of high dependence representation. To achieve the trade-off between structure complexity and classification accuracy, KDB allows to represent different number of interdependencies for different data sizes. The mRMR analysis and discriminative model selection have both previously been demonstrated to be computationally efficient approaches, the former improves the feature selection method and the latter improves the classification error of KDB. However, on the one hand, the mRMR analysis has not been studied in the context of KDB, and on the other hand, the discriminative model selection still can be improved more, such as removing the redundant dependencies in a BNC. Therefore, in this paper, we investigate the feasibility of applying discriminative model selection to remove redundant features and dependencies, and the interoperability of mRMR analysis and discriminative model selection.

Regular KDB utilizes mutual information between features and class variable to rank and sort all features first. Obviously, some independent features with high mutual information value may achieve higher rank but demonstrate weak conditional dependencies. However, the use of mRMR analysis makes up for this shortcoming. Moreover, KDB does not consider the negative effect caused by redundant features, which may bias the classification results. We use the discriminative model selection to achieve the aim of removing the redundant features and arcs in the Bayesian network.

We conduct experiments on 40 UCI datasets to explore the impact of a sorting method based on the mRMR criterion and discriminative model selection. The advantages of MKDB and MSKDB over KDB in terms of zero-one loss, respectively, demonstrate that each technique can help reduce KDB’s classification error. The advantages of MMKDB over KDB, MKDB and MSKDB further demonstrate that the interoperability of these two techniques is feasible. That is, there is strong synergy between the mRMR analysis and discriminative model selection in KDB and they can operate in tandem to reduce the classification error of KDB more effectively than does either in isolation. The fitting curve of goal difference between MMKDB and KDB clarifies the superior performance of the MMKDB on datasets of different scales. MMKDB not only retains the privileges of KDB, i.e., the capacity of high dependence representation and the model fitting ability on large datasets, but also improves the model fitting ability on small and medium datasets and enhances the classification efficiency on large datasets. These two techniques help save about 42% of KDB’s classification time and greatly improve the classification performance of KDB. Besides, we also have compared MMKDB against other state-of-the-art BNCs, such as NB, TAN and AODE. The results demonstrate that MMKDB achieves significant advantages over NB and TAN on medium and large datasets, and over AODE on large datasets in terms of classification performance. We additionally conduct a set of focused tests for some significance analysis, such as the Friedman test and the Nemenyi test. The results showed that the mean rank of MMKDB is significantly lower than those of NB, TAN, KDB, MKDB and MSKDB. MMKDB also achieves lower mean rank than AODE, but not significantly so.

## Figures and Tables

**Table 1:** Corresponding joint probability distributions of KDB when given different *k* values.

*k* Value	Joint Probability Distribution
k=1	$p\left(x_{1},\ldots ,x_{4},c\right)=p\left(c\right)p\left(x_{1}|c\right)p\left(x_{2}|x_{1},c\right)p\left(x_{3}|x_{1},c\right)p\left(x_{4}|x_{1},c\right)$
k=2	$p\left(x_{1},\ldots ,x_{4},c\right)=p\left(c\right)p\left(x_{1}|c\right)p\left(x_{2}|x_{1},c\right)p\left(x_{3}|x_{1},x_{2},c\right)p\left(x_{4}|x_{1},x_{2},c\right)$
k=3	$p\left(x_{1},\ldots ,x_{4},c\right)=p\left(c\right)p\left(x_{1}|c\right)p\left(x_{2}|x_{1},c\right)p\left(x_{3}|x_{1},x_{2},c\right)p\left(x_{4}|x_{1},x_{2},x_{3},c\right)$

**Table 2:** Space of approximate models of KDB with *n* feature subsets.

Feature Subsets	Joint Probability
$\left\{X_{1}\right\}$	$p{\left(x,c\right)}_{1}=p\left(c\right)p\left(x_{1}|c\right)$
$\left\{X_{1},X_{2}\right\}$	$p{\left(x,c\right)}_{2}=p\left(c\right)p\left(x_{1}|c\right)p\left(x_{2}|Pa\left(x_{2}\right),c\right)$
$\left\{X_{1},X_{2},X_{3}\right\}$	$p{\left(x,c\right)}_{3}=p\left(c\right)p\left(x_{1}|c\right)p\left(x_{2}|Pa\left(x_{2}\right),c\right)p\left(x_{3}|Pa\left(x_{3}\right),c\right)$
⋮	⋮
$\left\{X_{1},X_{2},X_{3},\ldots ,X_{n}\right\}$	$p{\left(x,c\right)}_{n}=p\left(c\right)p\left(x_{1}|c\right)p\left(x_{2}|Pa\left(x_{2}\right),c\right)p\left(x_{3}|Pa\left(x_{3}\right),c\right)\cdots p\left(x_{n}|Pa\left(x_{n}\right),c\right)$

**Table 3:** Datasets.

No.	Dataset	Inst	Feature	Class	No.	Dataset	Inst	Feature	Class
1	Contact-Lenses	24	4	3	21	Splice-C4.5	3177	60	3
2	Lung-Cancer	32	56	3	22	Hypo	3772	29	4
3	Labor	57	16	2	23	Sick	3772	29	2
4	Post-Operative	90	8	3	24	Abalone	4177	8	3
5	Zoo	101	16	7	25	Spambase	4601	57	2
6	Promoters	106	57	2	26	Waveform-5000	5000	40	3
7	Echocardiogram	131	6	2	27	Phoneme	5438	7	50
8	Autos	205	25	7	28	Page-Blocks	5473	10	5
9	Audio	226	69	24	29	Mushrooms	8124	22	2
10	Hungarian	294	13	2	30	Thyroid	9169	29	20
11	Dermatology	366	34	6	31	Pendigits	10,992	16	10
12	Horse-Colic	368	21	2	32	Sign	12,546	8	3
13	House-Votes-84	435	16	2	33	Nursery	12,960	8	5
14	Chess	551	39	2	34	Magic	19,020	10	2
15	Crx	690	15	2	35	Letter-Recog	20,000	16	26
16	Vehicle	846	18	4	36	Adult	48,842	14	2
17	Anneal	898	38	6	37	Shuttle	58,000	9	7
18	Led	1000	7	10	38	Connect-4	67,557	42	3
19	Volcanoes	1520	3	4	39	Localization	164,860	5	11
20	Car	1728	6	4	40	Census-Income	299,285	41	2

**Table 4:** W/D/L records when comparing the zero-one loss of MKDB and KDB.

W/D/L	KDB
MKDB	12/25/3

**Table 5:** W/D/L records when comparing the zero-one loss of MSKDB and KDB.

W/D/L	KDB
MSKDB	8/31/1

**Table 6:** W/D/L records when comparing the zero-one loss of KDB, MKDB, MSKDB and MMKDB.

W/D/L	KDB	MKDB	MSKDB
MMKDB	17/21/2	15/23/2	10/30/0

**Table 7:** W/D/L records in terms of zero-one loss: MMKDB vs. NB, TAN and AODE.

W/D/L	NB	TAN	AODE
MMKDB	26/5/9	24/9/7	20/9/11

## Appendix A

This appendix presents the detailed zero-one loss results of NB, TAN, AODE, KDB, MKDB, MSKDB and MMKDB in Table A1, and the ranks of different algorithms on 40 datasets are shown in Table A2.

**Table A1:** Detailed zero-one loss results of NB, TAN, AODE, KDB, MKDB, MSKDB and MMKDB.

Dataset	NB	TAN	AODE	KDB	MKDB	MSKDB	MMKDB
Contact-Lenses	0.3788	0.3788	0.3788	0.2946	0.2888	0.3713	0.3257
Lung-Cancer	0.4419	0.5997	0.5050	0.5050	0.6806	0.5259	0.5496
Labor	0.0355	0.0531	0.0531	0.0709	0.0347	0.0521	0.0343
Post-Operative	0.3478	0.3704	0.3366	0.3928	0.3758	0.3740	0.3692
Zoo	0.0300	0.0100	0.0300	0.0600	0.0490	0.0490	0.0387
Promoters	0.0763	0.1334	0.1334	0.3240	0.2896	0.3362	0.2582
Echocardiogram	0.3393	0.3315	0.3238	0.3315	0.3325	0.3325	0.3282
Autos	0.3153	0.2167	0.2069	0.2020	0.1931	0.1931	0.1859
Audio	0.2413	0.2949	0.2055	0.3486	0.2968	0.3022	0.2724
Hungarian	0.1615	0.1718	0.1684	0.2027	0.1953	0.2088	0.1928
Dermatology	0.0193	0.0331	0.0166	0.0690	0.0541	0.0676	0.0453
Horse-Colic	0.2196	0.2113	0.2031	0.2882	0.2475	0.1614	0.1620
House-Votes-84	0.0952	0.0558	0.0534	0.0488	0.0524	0.0478	0.0494
Chess	0.1136	0.0935	0.1008	0.0733	0.0737	0.0701	0.0727
Crx	0.1391	0.1493	0.1361	0.1639	0.1549	0.1478	0.1431
Vehicle	0.3963	0.2972	0.2925	0.2901	0.2809	0.2937	0.2784
Anneal	0.0383	0.0112	0.0090	0.0090	0.0097	0.0088	0.0087
Led	0.2697	0.2687	0.2707	0.2687	0.2633	0.2633	0.2599
Volcanoes	0.3349	0.3349	0.3349	0.3349	0.3283	0.3283	0.3240
Car	0.1414	0.0573	0.0824	0.0205	0.0201	0.0201	0.0198
Splice-C4.5	0.0448	0.0471	0.0369	0.1926	0.1786	0.0598	0.0590
Hypo	0.0139	0.0142	0.0096	0.0139	0.0118	0.0097	0.0091
Sick	0.0311	0.0260	0.0276	0.0219	0.0218	0.0213	0.0202
Abalone	0.4810	0.4633	0.4517	0.4647	0.4380	0.4555	0.4423
Spambase	0.1025	0.0676	0.0679	0.0663	0.0671	0.0652	0.0657
Waveform-5000	0.2026	0.1862	0.1477	0.2699	0.2584	0.2600	0.2503
Phoneme	0.2641	0.2760	0.2416	0.2075	0.2033	0.1890	0.1865
Page-Blocks	0.0625	0.0419	0.0341	0.0332	0.0341	0.0317	0.0313
Mushrooms	0.0198	0.0001	0.0001	0.0000	0.0000	0.0000	0.0000
Thyroid	0.1122	0.0727	0.0708	0.0726	0.0714	0.0707	0.0697
Pendigits	0.1193	0.0324	0.0202	0.0444	0.0444	0.0436	0.0438
Sign	0.3622	0.2783	0.2849	0.2311	0.2265	0.2265	0.2236
Nursery	0.0983	0.0661	0.0737	0.0182	0.0175	0.0178	0.0176
Magic	0.2261	0.1692	0.1770	0.1661	0.1618	0.1629	0.1597
Letter-Recog	0.2550	0.1313	0.0892	0.1028	0.0960	0.1008	0.0981
Adult	0.1608	0.1394	0.1508	0.1404	0.1183	0.1355	0.1343
Shuttle	0.0039	0.0015	0.0008	0.0008	0.0008	0.0008	0.0007
Connect-4	0.2811	0.2378	0.2444	0.2166	0.2197	0.2124	0.2168
Localization	0.5005	0.3611	0.3632	0.3097	0.2974	0.3035	0.2996
Census-Income	0.2387	0.0634	0.1014	0.0509	0.0506	0.0488	0.0483

**Table A2:** Ranks of different algorithms on 40 datasets.

Dataset	NB	TAN	AODE	KDB	MKDB	MSKDB	MMKDB
Contact-Lenses	5.5	5.5	5.5	1.5	1.5	5.5	3.0
Lung-Cancer	1.0	6.0	2.5	2.5	7.0	4.0	5.0
Labor	2.5	5.0	5.0	7.0	2.5	5.0	1.0
Post-Operative	2.0	3.0	1.0	7.0	6.0	5.0	4.0
Zoo	2.5	1.0	2.5	7.0	5.5	5.5	4.0
Promoters	1.0	2.5	2.5	6.0	5.0	7.0	4.0
Echocardiogram	6.0	2.5	1.0	2.5	6.0	6.0	4.0
Autos	7.0	6.0	5.0	4.0	2.5	2.5	1.0
Audio	2.0	4.0	1.0	7.0	5.0	6.0	3.0
Hungarian	1.0	3.0	2.0	6.0	5.0	7.0	4.0
Dermatology	2.0	3.0	1.0	6.5	5.0	6.5	4.0
Horse-Colic	5.0	4.0	3.0	7.0	6.0	1.0	2.0
House-Votes-84	7.0	6.0	4.5	1.5	4.5	1.5	3.0
Chess	7.0	5.0	6.0	2.0	4.0	1.0	3.0
Crx	2.0	4.0	1.0	7.0	6.0	5.0	3.0
Vehicle	7.0	5.0	4.0	3.0	2.0	6.0	1.0
Anneal	7.0	6.0	3.0	3.0	5.0	3.0	1.0
Led	6.0	3.5	7.0	3.5	3.5	3.5	1.0
Volcanoes	4.5	4.5	4.5	4.5	4.5	4.5	1.0
Car	7.0	5.0	6.0	3.0	3.0	3.0	1.0
Splice-C4.5	2.0	3.0	1.0	7.0	6.0	5.0	4.0
Hypo	5.5	7.0	2.0	5.5	4.0	3.0	1.0
Sick	7.0	5.0	6.0	3.0	4.0	2.0	1.0
Abalone	7.0	4.0	3.0	5.5	1.0	5.5	2.0
Spambase	7.0	4.0	5.0	1.0	6.0	2.0	3.0
Waveform-5000	3.0	2.0	1.0	7.0	5.0	6.0	4.0
Phoneme	6.0	7.0	5.0	3.5	3.5	2.0	1.0
Page-Blocks	7.0	6.0	4.0	3.0	5.0	2.0	1.0
Mushrooms	7.0	5.5	5.5	2.5	2.5	2.5	2.5
Thyroid	7.0	5.0	1.0	4.0	6.0	3.0	2.0
Pendigits	7.0	2.0	1.0	3.5	6.0	3.5	5.0
Sign	7.0	5.0	6.0	3.0	3.0	3.0	1.0
Nursery	7.0	5.0	6.0	3.5	1.0	3.5	2.0
Magic	7.0	5.0	6.0	3.5	2.0	3.5	1.0
Letter-Recog	7.0	6.0	1.0	4.5	2.0	4.5	3.0
Adult	7.0	4.0	6.0	5.0	1.0	3.0	2.0
Shuttle	7.0	6.0	3.5	3.5	3.5	3.5	1.0
Connect-4	7.0	5.0	6.0	1.5	4.0	1.5	3.0
Localization	7.0	5.0	6.0	3.5	1.0	3.5	2.0
Census-Income	7.0	5.0	6.0	3.0	4.0	2.0	1.0
Mean rank	5.34	4.53	3.73	4.20	4.00	3.83	2.39
